# The “Magical Theory” of AI in Medicine: Thematic Narrative Analysis

**DOI:** 10.2196/49795

**Published:** 2024-08-19

**Authors:** Giorgia Lorenzini, Laura Arbelaez Ossa, Stephen Milford, Bernice Simone Elger, David Martin Shaw, Eva De Clercq

**Affiliations:** 1 Institute for Biomedical Ethics University of Basel Basel Switzerland; 2 Unit for Health Law and Humanitarian Medicine Center for Legal Medicine University of Geneva Geneva Switzerland; 3 Health, Ethics and Society Universiteit Maastricht Maastricht Netherlands

**Keywords:** artificial intelligence, medicine, physicians, hype, narratives, qualitative research

## Abstract

**Background:**

The discourse surrounding medical artificial intelligence (AI) often focuses on narratives that either hype the technology’s potential or predict dystopian futures. AI narratives have a significant influence on the direction of research, funding, and public opinion and thus shape the future of medicine.

**Objective:**

The paper aims to offer critical reflections on AI narratives, with a specific focus on medical AI, and to raise awareness as to how people working with medical AI talk about AI and discharge their “narrative responsibility.”

**Methods:**

Qualitative semistructured interviews were conducted with 41 participants from different disciplines who were exposed to medical AI in their profession. The research represents a secondary analysis of data using a thematic narrative approach. The analysis resulted in 2 main themes, each with 2 other subthemes.

**Results:**

Stories about the AI-physician interaction depicted either a competitive or collaborative relationship. Some participants argued that AI might replace physicians, as it performs better than physicians. However, others believed that physicians should not be replaced and that AI should rather assist and support physicians. The idea of excessive technological deferral and automation bias was discussed, highlighting the risk of “losing” decisional power. The possibility that AI could relieve physicians from burnout and allow them to spend more time with patients was also considered. Finally, a few participants reported an extremely optimistic account of medical AI, while the majority criticized this type of story. The latter lamented the existence of a “magical theory” of medical AI, identified with techno-solutionist positions.

**Conclusions:**

Most of the participants reported a nuanced view of technology, recognizing both its benefits and challenges and avoiding polarized narratives. However, some participants did contribute to the hype surrounding medical AI, comparing it to human capabilities and depicting it as superior. Overall, the majority agreed that medical AI should assist rather than replace clinicians. The study concludes that a balanced narrative (that focuses on the technology’s present capabilities and limitations) is necessary to fully realize the potential of medical AI while avoiding unrealistic expectations and hype.

## Introduction

### Background

Artificial intelligence (AI) technologies are steadily emerging and intertwining with humans’ everyday lives and practices. Their applications are broad and diverse: in the field of health care, AI tools are supporting administrative tasks, predicting patients’ prognoses, monitoring health through wearable devices, reading computed tomography scans, accelerating drug discovery and development, and many more applications [1]. Particularly relevant for the present analysis are AI-enabled wearable devices (eg, smartwatches) and clinical decision support systems (CDSSs). CDSSs are AI-based tools that provide diagnostic and treatment suggestions based on patient data and test results [2,3]. They bear the potential to impact physicians’ clinical judgment, decision-making process, and their relationship with patients [4]. Lately, CDSSs are being combined with machine learning and deep learning techniques, thus generating hopes for faster and more accurate medical decisions and diagnoses [5]. Machine learning and deep learning are types of AI that continuously learn from the data they are fed [6]. Both wearables and CDSSs are artificial narrow intelligence as they are designed to perform only specific tasks. On the contrary, humans have general intelligence: they can excel in speech recognition, pattern recognition, decision-making, and creating. This is also the goal of AI research: with artificial general intelligence, the aim is to apply the same tool to different areas with similar satisfactory results and performance [7]. As artificial general intelligence is not currently a possibility, this paper focuses on artificial narrow intelligence applied in the medical context as CDSSs or wearable devices.

**Figure 1:**
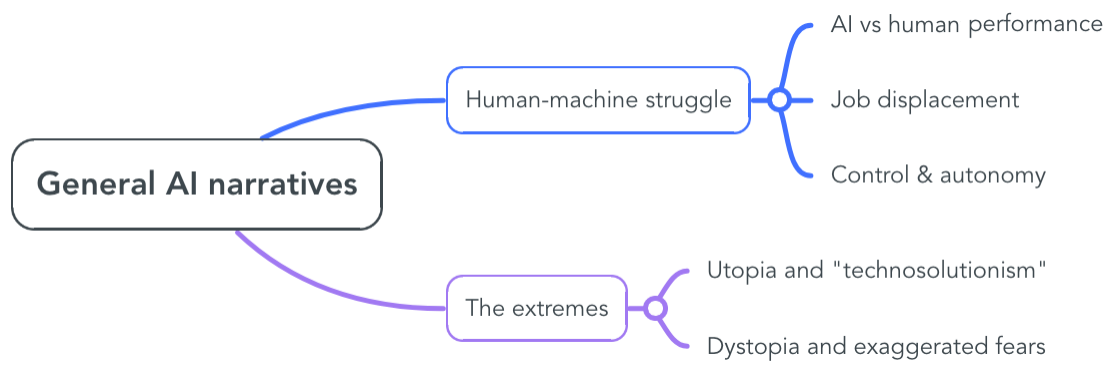
Summary of general artificial intelligence (AI) narratives identified in the literature and pertinent to this analysis.

Our work rests on 2 pillars: the first is medical AI, and the second is the creation and perpetuation of AI narratives by people exposed to AI in their profession. It is in the nature of humans to make sense of things, events, and situations. One way of doing this is through the construction of narratives that link together complex and multifaceted realities while assigning roles, identities, and values. Narratives are, therefore, stories we tell about our lives in a nuanced meaning-making effort [8]. It is important to analyze narratives because they reveal our attitudes, opinions, relationships, and emotions [9]. There is a multitude of general AI narratives (Figure 1), which come mainly from news outlets, science fiction accounts, the technology industry, and academic research. Prominent general AI narratives extensively concentrate on the struggle between humans and machines on different levels (ie, comparing their performances, worrying about job displacement, and wondering to which extent humans will relent control to AI). On the one hand, envisioning a world where AI takes over routine and tedious chores can be uplifting. On the other hand, it seems impossible to put to rest the underlying fear that it will take over everything else too, including more enjoyable and creative tasks [10]. Consequently, job displacement narratives are created based on the preoccupation that AI will render many jobs obsolete, particularly the ones revolving around menial tasks that could easily be automated [11]. This worry is exacerbated by the relentless comparison between humans’ and AI’s performances, as a means to validate AI’s capabilities [12]. In this human-machine struggle, AI is depicted as a superefficient tool at the service of a heartless capitalistic system [10]. At the same time, AI is appreciated exactly because it holds the potential to simplify humans’ lives: it is designed to help humans accomplish more with less effort. AI’s achievements are often publicly praised; this is continuously underlined when its performance excels humans’ capabilities. Accordingly, positive emotions and optimism are prevalent in social media posts about AI, also when the authors are experts in the field [13]. However, what is not acknowledged as much is that these successes are confined to very specific tasks: an AI that can excel in facial recognition will not automatically perform better than humans in driving cars. The lack of generalizability in AI means that human control and oversight are still pretty much needed. Having said that, narratives on AI taking control of human lives and societies are vastly popular [14]. What is usually incorrectly implied behind these narratives is that AI shares the human desire for greediness and its survival instincts, thus attributing these qualities to anthropomorphized machines [10,15].

Dominant AI narratives are often mistrusted or criticized in light of their extremism: they frequently depict either utopian or dystopian futures, light-years away from the complex and mundane reality, that misrepresent the present state of the technology [16]. For example, the way AI fails in the real world is far less epic and catastrophic from Hollywood conceptions: these failures usually happen when the AI does what it is programmed to do but with unintended consequences, that is, a robot trained to behave in ways that would meet humans’ approval pretending to be doing something useful [14,17]. The perpetuation of unrealistic AI failures inflates implausible fears while failing to address the real ways in which AI could fail [14]. The debate about AI is very polarized, and as opposed to apocalyptic predictions, there are overly optimistic accounts. The idea of AI being a “master technology” that would be able to unlock all sorts of useful technologies, including those that could help humanity achieve immortality, is common [18]. This leads to the imagination that AI could be considered a form of “holy grail” that bears the potential not only to provide for humanity’s needs but also to fulfill its wildest desires and dreams [18].

Narratives can have different functions for different authors and in different situations; in this analysis, the focus is on how narratives could influence medical AI development and uptake and particularly how they could foster a climate where medical AI supports physicians. Indeed, narratives on AI have the power to influence the further development of these technologies, the availability of funding, the directions of research, and the opinions and expectations of both experts and the public. They influence how new sociotechnical realities are accepted and address both the concerns and the hopes surrounding AI [19]. Therefore, they form the background against which AI is being developed, interpreted, and assessed [16]. While general AI narratives are widely studied and debated, particularly in the Western world [14,19-21], little data are available on AI applications in specific sectors. The lack of research on medical AI narratives, coupled with the perception of AI being particularly promising in the field of health care [22,23], calls for more attention to the topic. Humans have a “narrative responsibility” [24]: there is a duty to make sense of medical AI and to do it responsibly because these sensemaking processes concretely impact its development, implementation, and uptake. Since the stories humans tell about medical AI shape the future of health care, narratives cannot be conceived as normatively neutral. Narratives that support how we wish medicine to be for the years to come should be preferred [8].

### Objective

This paper offers a critical reflection on the existing literature on AI narratives. It is one of the first studies to examine the stories told by people who are professionally exposed to medical AI about its applications. This study compares these stories with the existing dominant general AI narratives so as to uncover meaningful similarities and differences. This study aims to raise awareness of how we talk about medical AI and how this can shape the future experiences of both patients and physicians. It is expected that some general AI narratives will be present in medical AI narratives. However, as this medical AI is implemented in a specific sector, namely, health care, with its particular features and challenges, some narratives will be unique for this context. The goal is to understand these similarities and differences to better evaluate medical AI narratives. Consequently, this study aims to recommend a more ethical approach when creating and perpetuating these narratives, considering their impact on physicians’ jobs and the physician-patient relationship.

## Methods

### Overview

The data used for this manuscript are part of a larger research project titled Ethical and Legal issues of Mobile Health-Data: Improving Understanding and Explainability of Digital Transformation and Data Technologies Using Artificial Intelligence (EXPLaiN), which aims to clarify the legal and ethical issues that need to be resolved for the collection, use, and analysis of health data with AI methods. The project is funded by the Swiss National Science Foundation. The first part of the study consisted of 41 semistructured interviews with participants who are exposed to medical AI. These participants were from a range of disciplines: medicine, philosophy, law, ethics, public health, and computer science. The interviews focused on the barriers and facilitators for the implementation of AI in clinical settings, particularly regarding CDSSs and wearable devices. The original study aimed to examine the current views, attitudes, knowledge, and barriers to using AI models in the analysis of health data and to support physicians and patients in their decision-making.

This analysis is a secondary analysis of these data and focuses on a subset of the data collected. While coding the data, it became apparent that narratives were often discussed. This justified a secondary analysis that was attentive to this aspect of the data. A second code tree was created based on the narratives identified in the literature, and the interviews were recoded. Of the 41 interviews, 30 (73%) were selected for the secondary analysis based on the presence of narrative elements about AI in health care. This selection, inherent to the secondary nature of the analysis, resulted in incomplete saturation in 1 subtheme, namely, “welcoming the holy grail.”

The data subset was analyzed using a thematic narrative approach that identified and reported stories participants told about medical AI [25,26]. This approach was chosen for its flexibility and ability to allow large data sets to be managed and reduced into themes [25]. The topic and the format of the data are not conducive to a structural narrative approach, as the narrative segments were relatively short and lacked common narrative characteristics (eg, characters with roles, a narrator, a complication, a resolution, and a coda) [27,28]. Therefore, a narrative thematic analysis was chosen, as it enabled single units of meaning, primarily phrases, and short paragraphs to be formed into themes and interpreted narratively [29]. With a narrative thematic approach, we could better describe how people exposed to AI in their profession experienced and understood medical AI, as well as how they made sense of it. This allowed for the analysis of underlying assumptions and values [27,30].

### Participants

Participants were purposively sampled and came from various disciplines and backgrounds: medicine, bioethics, public health, philosophy, psychology, economy, law, and computer science. Inclusion criteria, other than being exposed to medical AI in their profession, were the holding of a senior position, either in academia or in the private sector, hence excluding PhD students, interns, and junior professionals. Participants’ profiles were, for example, full professorship at a university, chief executive officer of a company working with AI, or a data protection officer at a hospital. Participants were recruited internationally; however, there was a focus on European and Swiss participants since the EXPLaiN project aimed to especially explore their attitudes. Participants were recruited because they were working with medical AI through projects, products, research, and development. Identification of participants occurred through publications or affiliations with companies working in the field of medical AI. Their email addresses were found on the web through their institution or company’s website. At the end of the interview, participants were asked if they knew someone meeting the inclusion criteria who would be interested in participating (snowball sampling).

First contact with participants was through email where they were invited to be interviewed by introducing the project and explaining the aims and the implications of their participation (eg, time commitment, voice recording, the method of transcription, and data pseudonymization format).

### Data Collection and Analysis

LAO and GL, who recruited the participants and conducted the one-on-one semistructured interviews, did not personally know the participants. LAO has a background in medicine and public health, while GL studied philosophy with a focus on ethics and philosophy of science. At the time of the data collection, both were PhD candidates in bioethics at the Institute of Biomedical Ethics of Basel.

Data were collected from November 2021 to April 2022 (therefore preceding some breakthrough such as ChatGPT; it could be hypothesized that after the most recent novelties in the AI field, such as natural language processing tools, narratives about AI might be different also in the health care sector) through semistructured interviews that lasted an average of 50 minutes. All the interviews of this subset were conducted on the web and recorded directly via Zoom (Zoom Video Communication, Inc). The original interview guide was composed of 13 questions, each with several prompts or follow-ups. The interview guide made use of 3 vignettes to better clarify and contextualize the questions. The questions were divided into 6 blocks: introductory questions (about the experience of the participant), general questions about using AI in medical practice, context-related questions about AI-patient relationships (vignette 1 involving a wearable device), context-related questions about physician-patient relationships with AI (vignette 2 involving CDSSs), context-related questions about private-public relationships (vignette 3), and closing questions. The more significant questions (reported in Textbox 1) for this analysis were questions numbered 3 and 4, as well as 3 prompts for question 8. However, relevant data were found elsewhere in the data set as the interviews were semistructured, and participants had some freedom in guiding the topics of the interview.

**Relevant questions and prompts from the interview guide.**

**Question numbers and questions**

- 3: I would like to start discussing clinical usability. What do you think about using artificial intelligence (AI) in clinical practice?
- 4: What would you consider the biggest challenges of using AI in health care?
- 8.6: How important it is that the physician understands AI?
- 8.8: Would AI have an impact on the physician-patient relationship?
- 8.9: Would AI challenge the traditional model of shared decision-making?

The interviews were transcribed verbatim by LAO, GL, and 2 students at the University of Basel using MAXQDA (VERBI GmbH), a software application designed to assist with qualitative analysis methods. LAO and GL checked all the transcripts and compared their correctness with the audio of the interviews. All data were securely stored on the server of the University of Basel and pseudonymized. Potentially reidentifiable information was removed from the transcripts.

After the original inductive coding, conducted equally by GL and LAO, GL reorganized the relevant coded sections for the secondary analysis. Upon consulting existing literature to identify dominant narratives, a new code tree was created, and the selected segments were deductively recoded. The selected data subset was interpreted through the lens of the existing categories of general AI narratives [31]. The new code tree composed the dominant AI narratives found in the literature. GL then selected the most significant codes and grouped them into themes.

### Ethical Considerations

All methods were approved by the Ethics Committee of Northwest and Central Switzerland, under Switzerland’s Human Research Act (HRA) Article 51 [32]. The methods were carried out in accordance with the relevant HRA guidelines and regulations. After revision, the Ethics Committee of Northwest and Central Switzerland concluded that interviewing AI professionals falls outside the HRA and requires only verbal consent at the beginning of an interview (declaration of no objection AO_2021-00045).

All personal data were pseudonymized and safely stored on the server at University of Basel. The key is accessible only to the research team. Potentially reidentifiable data were omitted from publication. No compensation was offered to participants.

## Results

### Overview

For this analysis, we used 30 interviews and reported at least 1 quote from each. We selected this subset because narratives were not prominent in all interviews. It was challenging to categorize participants into disciplines, as AI is notoriously an interdisciplinary field. More often than not, participants had mixed backgrounds and were dealing with medical AI from different points of view. In categorizing participants, we picked their main expertise: 9 (30%) participants had a background in medicine, 6 (20%) in bioethics, 6 (20%) in law, 3 (10%) in computer science, 2 (7%) in public health, 2 (7%) in philosophy, 1 (3%) in psychology, and 1 (3%) in economy. The vast majority of the selected participants (21/30, 70%) were male (female participants: n=9, 30%). Only 5 (17%) participants were located outside Europe: 3 (10%) in the United States, 1 (3%) in Canada, and 1 (3%) in South Africa (for more details on the participants, please refer to the Multimedia Appendix 1).

**Figure 2:**
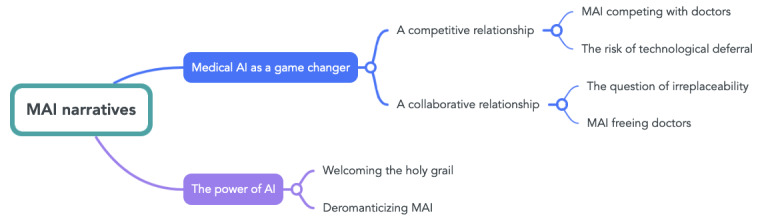
Themes and subthemes that emerged from the thematic narrative analysis. AI: artificial intelligence; MAI: medical artificial intelligence.

Our analysis identified 2 main themes and various subthemes (Figure 2). Representative anonymized quotes were taken from the interviews to illustrate the reported results. Participants are identified with the abbreviation of their main expertise and a number: bioethics (BE), computer science (CS), economy (EC), law (LW), medicine (ME), public health (PH), philosophy (PL), and psychology (PS).

### Medical AI as a Game Changer

#### Overview

With regard to physicians and medical AI relationships, attitudes fell into 2 main groups. Some participants depicted a rather competitive relationship and compared the performances and capabilities of medical AI to those of physicians. The majority, however, emphasized how AI can support clinicians, thus outlining a more collaborative relationship and focusing on the benefits of this cooperation. Nevertheless, these 2 groups shared the underlying idea that AI would be a game changer for medicine, and both emphasized how it could be useful in health care.

#### A Competitive Relationship

##### Medical AI Competing With Physicians

Some participants described AI as a competitor to physicians and argued that not only clinicians are dependent on AI but also they could even be replaced by it. Medical AI was said to outperform clinicians in pattern recognition and data processing. AI was believed to notice aspects that physicians would miss, hence emphasizing the limitations of human capabilities and describing AI as being faster, more accurate, and less costly than human physicians:

AI is able to grasp so many ideas within a very small time interval [and] also integrate information that physicians might even oversee that this might be even like more precise than physicians. And I think this is also an advantage.

ME7

The AI tool uncovers a pattern that the clinician did not pick up or maybe could not have picked up within a human limited abilities.

BE5

[Medical AI is] very inexpensive to use. In principle, like once you’ve trained the system for let’s say a diagnosis, you can basically use these things on a regular laptop or smartphone even..... It doesn’t come for free, but it is rather inexpensive and easy to get.

BE4

Comparing physicians and AI performance, abilities, and costs sometimes resulted in claims about the obsolescence of physicians since AI would be better in many aspects of a physician’s role, while also being faster and cheaper, and it seemed to be preferable to delegating tasks to AI. It was rarely implied that physicians as a whole would be replaced. More commonly, it was suggested that some specific tasks could be carried out by AI. A common limitation was that AI could not interact with patients as at present it lacked the necessary skills. Presuming that AI capacities would steadily improve, a few participants wondered whether in the future medical AI might be able to assume all physicians’ duties:

Nowadays there are certain things that might not be outsourced to machines in terms of human interactions. But on the other side, I think, if we wait long enough, we can basically outsource everything to machines.

PH1

I’m pretty sure that the physician will be quite cautious, at least in the beginning, when they know that they use these kinds of products [medical AI], but maybe with time, you know, when they are used to it, in like 5 years, 10 years, 15 years, maybe with time they could lose probably autonomy.

LW6

You will actually have better outcomes if you don’t involve humans.

ME9

Things were different for image recognition: several participants mentioned that medical AI gave outstanding results in radiology. This led to the possibility of outsourcing routine cases to AI while consulting radiologists only for peculiar cases:

I think one of the big places where it’s already implemented is in radiology. Meaning, recognition of patterns in pictures; machines are better at it than we are.

ME1

What people have been doing in radiology, I think it’s also awesome.... The machine can give you feedback right away and maybe you just use the humans for very specific cases.

CS1

And as more people use that tool, there might be the temptation that therefore maybe we don’t need as many dermatologists. Or as many specialists in certain areas, like radiology. Because we have very good AI that is able to detect cancer from X-rays. Or covid from X-rays of lungs.

PS1

##### The Risk of Technological Deferral

While pondering the idea of a more autonomous medical AI, many participants worried about the risks of excessive technological deferral (giving too much power to technology). Automation bias, namely, the tendency to overrely on automatic decision-making tools, was mentioned as an issue in areas of practice that are time-sensitive:

In the long run [physicians] end up with them just following what the machine says.

PL1

There is a very real risk, especially in areas of practice that have time pressure, that we will see automation bias, that we will see AI systems that formally were advisory, actually being the ones who decide treatment choices.

BE2

This tension on who holds the final decision-making responsibility was framed as an actual conflict, with potentially detrimental consequences if the humans were to “lose” their decision-making power. Physicians might also be intimidated by this outstanding tool and therefore would struggle to override its decisions even when they did not agree with it:

Can you even win, so to speak? So, that might be the bigger danger, where you say like “well, the machine says that, so therefore it is correct.”

PL2

The recurrent mentioned consequences of deferring decision-making powers to medical AI were dependency on technology, with fewer and fewer specialists trained and a gradual loss of autonomy for physicians. Many participants in this group worried about physicians’ autonomy being endangered by medical AI and described the technology as authoritarian or tyrannical:

Well, if the algorithms prove to be better than physicians then you would have to change the role of physicians from decision-makers to more just like people, in the end, giving injections.

CS2

#### A Collaborative Relationship

##### The Question of Irreplaceability

For many participants, physicians are not to be replaced by AI; rather, AI enables them and supports their daily practice and decision-making activities:

It should go in the direction that the systems are not seen as a competitor to the physicians but more as a cooperation between both. And I think what it’s worthwhile, what it’s important, it’s that the cooperation leads to better results.

EC1

The use of technology is going to assist the physician and not harm because in the end it’s called a clinical decision support tool, not a clinical decision maker tool.

ME3

But I think it will never, never replace the main diagnosis of a physician. So, this will always be a support tool. Which has to be as well validated beforehand.

LW2

We need to be clear that AI is not just coming along to replace physicians and when they go to the GP [general practitioner], they’re going to see a robot instead and the robot won’t understand anything about them and it’ll just give them a stamp prescription that is the same as everyone else. That’s not what AI is. And certainly not in the next few decades, will it be used for anything other than decision support.

ME5

Some interviewees noted how humanity is irreplaceable, while others described medical AI as an assistive tool that is not designed to replace physicians but to empower them. Participants in this group emphasized the idea of medical AI “assisting,” “helping,” “empowering,” and “supporting” clinicians rather than comparing their ability, accuracy, and cost.

When emphasizing physicians’ irreplaceability, participants referred to the sensibility, emotivity, and empathy that are needed in medical decision-making. Given the current state of the art, medical AI is unable to grasp the complex totality of the patient’s situation. Many participants also questioned patients’ willingness to relinquish the physician-patient relationship in favor of an AI-patient relationship. They argued that communication with AI would not be authentic, as it would not consider patients’ personhood. Therefore, these participants concluded that medical AI should never override physicians’ decisions; rather, it should promote and preserve physicians’ agency:

The patient needs a person he can talk [to], a person that can read their emotions, feelings.

LW4

I think medicine has a certain degree of nuances, that only a person might catch and not a computer. And you can’t let these computers or AI run autonomously.

ME4

Independently from AI capabilities (whether it outperforms physicians or not and whether it is limited or not), physicians remain essential: medical AI should always be considered in the light of physicians’ clinical judgment and never left unsupervised. According to this description of medical AI, physicians should always keep an active role in decision-making:

I think the one who has the responsibility to make decisions is, or will always be, the physician.

LW4

I expect that the technology will help you give an assessment, but that you will still have a clinician that will evaluate further that kind of technical assessment by software. So it’s not fully replacing an intervention as such. It’s helping, supporting a development.

LW5

I think that the physician has to make a decision based on their training. That’s their responsibility.

ME8

##### Medical AI Freeing Physicians

The collaborative relationship narrative does not depict physicians as dependent on their tools; rather, it suggests that medical AI could constitute an important resource. The relationship is described as a fruitful partnership, and the outcome would be a general improvement to both physicians’ practice and their work conditions. Medical AI could free physicians from burdensome tasks, hence relieving them from burnout and allowing them to spend more quality time with patients:

[Medical AI] could improve the physician-patient communication.... So I am kind of hoping that, in that way, because of AI certain aspects of healthcare could be simplified and automated, but that equally should generate room for more empathy between physicians and patients.

PS1

[Medical AI] is helping physicians to really focus on, or be able to have more time for patients and less to spend with tools.

CS3

What I hope it’ll do it’s improve the relationship between the patient and the physician. What I mean by this is [that] the physician is going to be relieved from the burnout.

ME3

### The Power of Medical AI

#### Overview

Most of the participants were optimistic about the future of medicine when AI was involved and reported an overall positive impact, or potential, of this technology. While a large part of the answers balanced medical AI’s advantages with the challenges it introduces, some focused only on the benefits of the technology. At the same time, many interviewees identified a hype-type narrative of medical AI and problematized it. In this context, hype is understood as an exaggeratedly optimistic rhetoric about an emerging technology [33].

#### Welcoming the Holy Grail

In a few interviews, medical AI was discussed mainly in positive terms. These participants did not see any negative aspects or concerns about the technology. Medical AI was deemed always useful, and if it was not useful for something yet, it surely would be in the future. It encapsulated so many opportunities for health care that 1 participant referred to it as “the holy grail.” Consequently, medical AI was expected to solve a wide range of problems:

I basically don’t see any negative effects, like, I can’t really see any negative effects.

LW1

So, it seems to me that it’s both inevitable and good that we have it [medical AI].

BE2

What do you think about using AI or machine learning in clinical practice? [GL] I think it’s the Holy Grail.

CS1

Well, it [medical AI]’s a game changer. And I think that our wild dream about getting personalized medicine is really at hand.

LW3

#### Medical AI Is Not Magical

A significant part of the participants addressed the romanticization of this technology and highlighted the importance of promoting a more truthful narrative. “Truth” and “reality” were terms often mentioned when discussing the medical AI hype: it was deemed untruthful, unhelpful, and unrealistic, and this was judged problematic:

The problem is that this enthusiasm is so uncritical and then we build into this. This is not giving us the truth and not helping us to generate probabilities. This is the problem that I hugely see.

BE3

According to the participants, one of the consequences of the hype around medical AI is that it is impossible to live up to the expectations that it builds. Therefore, some participants were profoundly critical toward overhyped accounts of the capabilities of medical AI:

There is so much hype in this field [medical AI] and this builds narratives and expectations. And to live up to those expectations is always challenging.

ME2

So that has, probably now backwards looking, not been so clever to phrase it as the silver bullet solution to everything, to patient autonomy, or patient empowerment, to more efficient and better healthcare.

BE1

The outcome of this ideology is that medical AI is portrayed as the appropriate means to tackle every pressing issue of health care: AI is the hammer that fixes everything. Techno-solutionist narratives would misunderstand AI and promote a representation of the technology as if it were some kind of magical tool:

The hype around the technology at the moment, you know, that people think that it can solve everything. It’s like they have a hammer and everything is a nail.

PH2

I think a lot of people and a lot of physicians kind of have the magical theory of machine learning, where you just kind of throw the numbers in the hopper, shake it out, and you get the results by magic.

BE6

The major problem of deep learning today are the people doing deep learning because they think they will solve everything with that and the ignorant people because they don’t understand what is deep learning and they think it’s magic that will solve everything.

ME6

## Discussion

### Principal Findings

The accounts of medical AI that emerged from these interviews are more realistic and less influenced by science fiction narratives than the general discourse on AI. Dystopian futures were not reported, and only a few participants described AI as a utopian technology that would address all challenges faced by the health care sector. While general AI narratives are usually polarized, describing AI as either the milestone of a better future for humanity or the cause of all evils [19,34,35], study participants often found a middle path between medical AI’s promises and risks, thus avoiding alignment with extreme positions and providing instead a more nuanced depiction of the technology. We hypothesize that people exposed to AI in their profession are less prone to exaggerated and polarized narratives, while lay people tend to be more susceptible to these narratives as they feel they have less control over the technology [36]. The lack of a strongly polarized discourse in medical AI can be regarded as positive: the contradictions present in narratives that are diametrically opposed and irreconcilable hinder a nuanced and sophisticated understanding of the technology [21].

However, our study sample was not exempt from hype narratives that uncritically focused on the expected benefits of medical AI. This confirmed the existence of hype narratives, which are already reported in the literature as well as the conceptualization of AI as a “holy grail” technology [22,23,34].

Claims about superiority are very popular in AI narratives, not only in fictional and media narratives but also in the scientific discourse, as researchers frequently compare AI with humans’ capabilities and performances as a means of validating the technology [12,37]. The physician-AI juxtaposition ends with depicting the classical human-machine struggle panorama, where physicians are menaced by an authoritarian machine that outperforms them and that leaves humans dependent on it, no longer in control, and stripped of their agency [12,14,19,35]. Indeed, 1 participant described this struggle as a real win-lose situation.

While a few participants hyped medical AI, the majority recognized both the advantages and the challenges introduced by AI in health care. Therefore, stronger than the hype narrative were the cautionary tales of avoiding a “myopic techno-solutionism” and the criticism of this hype [34]. Techno-solutionism is the ideology in which every kind of problem (technical, social, economic, political, psychological, or physical) can be ameliorated with an “appropriately designed” technological solution [38]. Attributing magical properties to AI, meaning that it can somehow address every problem, reveals a shallow understanding of the technology. This requires better education, which can be achieved through the establishment of a more balanced narrative that realistically assesses medical AI’s current capabilities and shortcomings [37].

Participants confirmed the idea that medical AI narratives can sometimes be detached from the everyday reality of the technology and that the hyping of AI leads to unrealistic expectations and overpromising while obscuring technological bottlenecks [19,21]. Therefore, our findings demonstrate that the current dominant narratives can mislead the understandings of medical AI, even in people working with it. Instead, “narratives should focus on the realities of AI’s present capabilities” [34] and take into account the narrative responsibility that is always entailed when the future of medicine with AI is imagined. Every story we tell about medical AI shapes its development, adoption, and perception in health care in ways that are not normatively neutral.

Accordingly, almost all participants recognized the limitations of AI. There is a risk that by failing to acknowledge the potential problems and shortcomings of medical AI, the hype narrative might further exacerbate these hidden specters. The need for a more realistic narrative that returns the image of the actual state of the art is commonly present both in the interviews and in the debate about AI narratives [14,19,34].

With the exception of a few participants, there was a general agreement that AI could not and would not replace human clinicians. This finding is present in the literature about the future of medicine with AI; for example, patients appeared less prone to seek medical assistance if AI provides it, even if it was better than a human expert [39]. When it comes to this topic, there is an alignment between different narratives that appear to share similar moral codes according to which medical AI cannot entirely replace the physician’s role or human interaction [40]. Therefore, this could be regarded as the “proper narrative” of the AI-physician relationship, and, as such, it might take the form of a collective narrative or “imaginary,” judged true without a need for further justification [41]. The prevailing idea remains: “patients will always need human physicians” [42].

Having determined that medical AI is to assist clinicians, it remains to be assessed whether it will have an impact on the physician-patient relationship. Some participants believed that medical AI would ameliorate their relationships, for example, by allowing physicians to spend more time with patients. This is also a popular idea in the literature to the extent that some claim that medical AI could be an opportunity to make physicians more human and empathetic [43-47]. However, as with many things about AI, opinions are divided, and this idea is also widely criticized. It could be that physicians will visit more patients in the time AI saved, thus maintaining the status quo or worsening care provisions [12,48,49]. Consequently, medical AI might not necessarily have a positive impact on the physician-patient relationship as either the participants in our study or many prominent voices in research think.

### Limitations

There is a clear prevalence of a Western perspective in our study. Hence, it remains questionable whether our findings are valid in other contexts.

The interview guide that we used for this study focused on certain applications of AI in medicine, namely, CDSSs and wearable devices (eg, smartwatches). This may have limited the discourse on possible outcomes and futures. Moreover, question 8.8. discusses the “traditional” model of shared decision-making; this wording could be considered nonneutral and leading.

Before commencing this analysis, we have conducted theoretical research on the ethical issues of medical AI. This led us to publications where we took a position on the role of AI in health care and the physician-patient relationship. We concluded that medical AI is currently, and should continue to be, an assistive tool that should support physicians’ and patients’ decision-making. We acknowledge that this belief was already sedimented at the time of data collection and analysis, thus possibly shaping the way in which we presented the results.

### Conclusions

Through the establishment of a more realistic and nuanced medical AI narrative, it is easier to describe AI tools as assistive. The discourse about their benefits, risks, and possible applications is less spectacular. Narratives that support the idea of AI augmenting humans’ capabilities, rather than substituting them, should be preferred as these narratives better correspond to the current reality of the technology [34]. It is also fundamental to raise awareness of the narrative responsibility that humans have to make sense of, interpret, and narrate medical AI in a way that shapes a positive future for medicine. Similarly, humans are responsible for scrutinizing the dominant narratives and evaluating them [24]. Everyone has this responsibility when talking about medical AI, including researchers, since we all can impact the future of technology, although to different degrees. Failing to exercise this narrative responsibility would entail relinquishing our sense-making task to other narrators (eg, big tech, transhumanists, governments, etc). The consequence would be a world in which we live in the narrative created by others for us. This world would be one in which the majority of humanity delegated the construction of our future to a few, in that they did not participate in the process that would shape what mattered most in the present [24,50].

Disproportionate fears and expectations could halt the development of medical AI, for example, by generating opposition or disillusionment when the technology does not live up to its promised expectations [19,21]. Medical AI narratives shape the role of AI in societies in ways that are ethically and politically relevant and can influence the perceptions of citizens, policy makers, politicians, health care personnel, and researchers [8,16]. Therefore, narratives have a constitutive role that is more than strictly descriptive: it is performative. Narratives have the power to decide the future of medical AI [51,52]. We argue that it is important to recognize the role that narratives of technologies play for humanity and reflect on which type of narrative is dominant in medical AI. This is a fundamental ethical issue that cannot be overlooked. It must be addressed so as to shape our desired future for medicine.

## Appendix

Multimedia Appendix 1 Participants’ characteristics.

## Abbreviations

AI: artificial intelligence
CDSS: clinical decision support system
EXPLaiN: Ethical and Legal issues of Mobile Health-Data: Improving Understanding and Explainability of Digital Transformation and Data Technologies Using Artificial Intelligence
HRA: Human Research Act

## Data Availability

The data sets generated during and analyzed during this study are available from the corresponding author on reasonable request.

## References

[1] Basu K, Sinha R, Ong A, Basu T (2020). Artificial intelligence: how is it changing medical sciences and its future?. Indian J Dermatol.

[2] Sutton RT, Pincock D, Baumgart DC, Sadowski DC, Fedorak RN, Kroeker KI (2020). An overview of clinical decision support systems: benefits, risks, and strategies for success. NPJ Digit Med.

[3] Berner ES, La Lande TJ, Berner E (2007). Overview of clinical decision support systems. Clinical Decision Support Systems: Theory and Practice.

[4] Lorenzini G, Arbelaez Ossa L, Shaw DM, Elger BS (2023). Artificial intelligence and the doctor-patient relationship expanding the paradigm of shared decision making. Bioethics.

[5] Wang D, Wang L, Zhang Z, Wang D, Zhu H, Gao Y, Fan X, Tian F (2021). “Brilliant AI doctor” in rural clinics: challenges in AI-powered clinical decision support system deployment. Proceedings of the 2021 CHI Conference on Human Factors in Computing Systems.

[6] Du Y, McNestry C, Wei L, Antoniadi AM, McAuliffe FM, Mooney C (2023). Machine learning-based clinical decision support systems for pregnancy care: a systematic review. Int J Med Inform.

[7] Kaplan A, Haenlein M (2019). Siri, Siri, in my hand: who’s the fairest in the land? On the interpretations, illustrations, and implications of artificial intelligence. Bus Horiz.

[8] Coeckelbergh M (2021). Time machines: artificial intelligence, process, and narrative. Philos Technol.

[9] Wertz FJ, Charmaz K, McMullen LM, Josselson R, Anderson R, McSpadden E (2011). Five Ways of Doing Qualitative Analysis: Phenomenological Psychology, Grounded Theory, Discourse Analysis, Narrative Research, and Intuitive.

[10] Dihal K (2020). Enslaved minds: artificial intelligence, slavery, and revolt get access arrow. AI Narratives: A History of Imaginative Thinking about Intelligent Machines.

[11] Guenduez AA, Mettler T (2023). Strategically constructed narratives on artificial intelligence: what stories are told in governmental artificial intelligence policies?. Gov Inf Q.

[12] Ostherr K (2022). Artificial intelligence and medical humanities. J Med Humanit.

[13] Manikonda L, Kambhampati S (2017). Tweeting AI: perceptions of lay versus expert Twitterati. Proceedings of the International AAAI Conference on Web and Social Media.

[14] Recchia G, Cave S, Dihal K, Dillon S (2020). The fall and rise of AI: investigating AI narratives with computational methods. AI Narratives: A History of Imaginative Thinking about Intelligent Machines.

[15] Singler B, Cave S, Dihal K, Dillon S (2020). Artificial intelligence and the parent–child narrative. AI Narratives: A History of Imaginative Thinking about Intelligent Machines.

[16] Cave S, Dihal K, Dillon S, Cave S, Dihal K, Dillon S (2020). Introduction: imagining AI. AI Narratives: A History of Imaginative Thinking about Intelligent Machines.

[17] Christiano P, Leike J, Brown TB, Martic M, Legg S, Amodei A Deep reinforcement learning from human preferences. https://arxiv.org/abs/1706.03741.

[18] Cave S, Cave S, Dihal K, Dillon S (2020). AI: artificial immortality and narratives of mind uploading. AI Narratives: A History of Imaginative Thinking about Intelligent Machines.

[19] Cave S, Craig C, Dihal K, Dillon S, Montgomery J, Singler B, Taylor L (2018). Portrayals and perceptions of AI and why they matter. The Royal Society.

[20] Cave S, Dihal K (2020). The whiteness of AI. Philos Technol.

[21] Vicsek L (2020). Artificial intelligence and the future of work – lessons from the sociology of expectations. Int J Sociol Soc Policy.

[22] Cameron D, Maguire K (2017). Public views of machine learning: digital natives. The Royal Society.

[23] Frost EK, Carter SM (2020). Reporting of screening and diagnostic AI rarely acknowledges ethical, legal, and social implications: a mass media frame analysis. BMC Med Inform Decis Mak.

[24] Coeckelbergh M (2021). Narrative responsibility and artificial intelligence: how AI challenges human responsibility and sense-making. AI Soc.

[25] McAllum K, Fox S, Simpson M, Unson C (2019). A comparative tale of two methods: how thematic and narrative analyses author the data story differently. Commun Res Pract.

[26] Braun V, Clarke V (2006). Using thematic analysis in psychology. Qual Res Psychol.

[27] Bruner J, Beilin H, Pufall PB (1992). The narrative construction of reality. Piaget's Theory: Prospects and Possibilities.

[28] Clarke V, Braun V (2016). Thematic analysis. J Posit Psychol.

[29] Ross JA, Green C (2010). Inside the experience of anorexia nervosa: a narrative thematic analysis. Couns Psychother Res.

[30] Fisher WR (2009). Narration as a human communication paradigm: the case of public moral argument. Commun Monogr.

[31] Braun V, Clarke V (2022). Conceptual and design thinking for thematic analysis. Qual Psychol.

[32] (2011). Federal Act on research involving human beings (Human Research Act, HRA), chapter 9: research ethics committees. Fedlex.

[33] van Lente H, Spitters C, Peine A (2013). Comparing technological hype cycles: towards a theory. Technol Forecast Soc Change.

[34] Chubb J, Reed D, Cowling P (2022). Expert views about missing AI narratives: is there an AI story crisis?. AI Soc.

[35] Klarmann N Artificial intelligence narratives: an objective perspective on current developments. https://arxiv.org/abs/2103.11961.

[36] Borup M, Brown N, Konrad K, Van Lente H (2006). The sociology of expectations in science and technology. Technol Anal Strateg Manag.

[37] Park SH, Do KH, Kim S, Park JH, Lim YS (2019). What should medical students know about artificial intelligence in medicine?. J Educ Eval Health Prof.

[38] Gardner J, Warren N (2019). Learning from deep brain stimulation: the fallacy of techno-solutionism and the need for 'regimes of care'. Med Health Care Philos.

[39] Longoni C, Bonezzi A, Morewedge CK (2019). Resistance to medical artificial intelligence. J Consum Res.

[40] Berkhout F (2006). Normative expectations in systems innovation. Technol Anal Strateg Manag.

[41] Konrad K (2006). The social dynamics of expectations: the interaction of collective and actor-specific expectations on electronic commerce and interactive television. Technol Anal Strateg Manag.

[42] Mittelman M, Markham S, Taylor M (2018). Patient commentary: stop hyping artificial intelligence-patients will always need human doctors. BMJ.

[43] Topol E (2019). Deep Medicine: How Artificial Intelligence Can Make Healthcare Human Again.

[44] Verghese A, Shah NH, Harrington RA (2018). What this computer needs is a physician: humanism and artificial intelligence. JAMA.

[45] Nundy S, Montgomery T, Wachter RM (2019). Promoting trust between patients and physicians in the era of artificial intelligence. JAMA.

[46] Mittelstadt B The impact of artificial intelligence on the doctor-patient relationship. Council of Europe.

[47] Liu X, Keane PA, Denniston AK (2018). Time to regenerate: the doctor in the age of artificial intelligence. J R Soc Med.

[48] Sauerbrei A, Kerasidou A, Lucivero F, Hallowell N (2023). The impact of artificial intelligence on the person-centred, doctor-patient relationship: some problems and solutions. BMC Med Inform Decis Mak.

[49] Sparrow R, Hatherley J (2020). High hopes for "deep medicine"? AI, economics, and the future of care. Hastings Cent Rep.

[50] Sanz Menéndez L, Cabello C (2000). Expectations and learning as principles of shaping the future. Unidad de Políticas Comparadas (CSIC).

[51] Guice J (1999). Designing the future: the culture of new trends in science and technology. Res Policy.

[52] van Lente H (2012). Navigating foresight in a sea of expectations: lessons from the sociology of expectations. Technol Anal Strateg Manag.

