# Supplementary material for: The “Magical Theory” of AI in Medicine: Thematic Narrative Analysis
Source: JMIR AI. 2024 Aug 19;3:e49795. doi: 10.2196/49795 (PMC11369530; doi:10.2196/49795)
Supplement: Multimedia Appendix 1 [file ai_v3i1e49795_app1.docx]

| **Research participant** | **Country** | **Sex** | **Expertise** |
| --- | --- | --- | --- |
| ME1 | Africa | Male | Medicine |
| ME2 | Europe | Male | Medicine |
| ME3 | North America | Male | Medicine |
| ME4 | North America | Male | Medicine |
| ME5 | Europe | Female | Medicine |
| ME6 | Switzerland | Male | Medicine |
| ME7 | Europe | Female | Medicine |
| ME8 | Switzerland | Male | Medicine |
| ME9 | Switzerland | Female | Medicine |
| BE1 | Europe | Female | Bioethics |
| BE2 | Europe | Male | Bioethics |
| BE3 | Switzerland | Female | Bioethics |
| BE4 | Europe | Male | Bioethics |
| BE5 | Europe | Male | Bioethics |
| BE6 | North America | Female | Bioethics |
| LW1 | Switzerland | Male | Law |
| LW2 | Switzerland | Female | Law |
| LW3 | Europe | Male | Law |
| LW4 | Switzerland | Male | Law |
| LW5 | Europe | Male | Law |
| LW6 | Switzerland | Male | Law |
| CS1 | Europe | Male | Computer science |
| CS2 | Switzerland | Male | Computer science |
| CS3 | Europe | Male | Computer science |
| PH1 | Switzerland | Female | Public health |
| PH2 | Europe | Female | Public health |
| PL1 | Europe | Male | Philosophy |
| PL2 | North America | Male | Philosophy |
| PS1 | Europe | Male | Psychology |
| EC1 | Europe | Male | Economy |
